# The psychological mechanism of basic psychological need frustration affecting job burnout: a qualitative study from China

**DOI:** 10.3389/fpsyg.2024.1400441

**Published:** 2024-10-29

**Authors:** Hairong Shi

**Affiliations:** School of Biology and Food Engineering, Chuzhou University, Chuzhou, China

**Keywords:** job burnout, basic psychological needs, need frustration, consequences of frustration, college counselors

## Abstract

**Introduction:**

Job burnout is a common issue in most professions, and it can have adverse effects on employees, their families, clients, and organizations. It is essential to address and resolve job burnout syndrome. More research is needed to understand the underlying psychological mechanisms involved in job burnout. This study introduces the concept of primary psychological need frustration to explore its impact on the psychological processes involved in job burnout.

**Methods:**

This study adopted a qualitative research methodology based on purposive sampling and convenience sampling principles. Eight grassroots senior counselors with over 13 years of teaching experience at a Chinese university were selected as the study cases. The data were gathered through semi-structured interviews and analyzed thoroughly via cluster analysis, which involved examining the text data word by word, sentence by sentence, line by line, and fragment by fragment. NVivo 11 software was used to register and code the text data.

**Results:**

The study revealed that the subjects experienced high levels of frustration with their basic psychological needs. This frustration was evident in the coexistence of negative job characteristics and a lack of autonomy, a hostile professional environment and a lack of competence, and the negative behavior of others and relationship frustration. The study also revealed that the four types of primary psychological need frustration were strongly linked to job burnout: A lack of control motivation or motivation, the pursuit of external goals, negative behavior patterns, and the causal orientation of a controlled style. These factors positively predicted various dimensions of job burnout and positively affected the frustration of basic psychological needs.

**Conclusion:**

This study effectively explains the psychological process behind why individuals experience severe job burnout in a controlled organizational environment due to frustration with basic psychological needs. This study also highlights the internal causal relationship between primary psychological needs, frustration, and job burnout. This insight can help employees and organizations prevent and detect early job burnout syndrome and enhance employees’ occupational well-being and organizational vitality.

## 1 Introduction

The concept of job burnout was first proposed by Freudenberger (1974), an American clinical psychologist, in 1974. He believed that job burnout was a state of exhaustion caused by high work intensity, long working hours, and disregard for one’s own needs. According to Cherniss (1980), job burnout is an attitude and behavior expressed in a negative way under work pressure. According to Pines and Aronson (1988), job burnout is a symptom of emotional and energetic exhaustion in a long-term emotional work situation. Brill (1984) believes that job burnout is a dysfunctional state. According to Lee and Ashforth (1993), job burnout is an emergency response to work stress. Maslach and Jackson (1981), an American social psychologist, defined “job burnout” as “a symptom of emotional exhaustion, depersonalization and low personal accomplishment of individuals in the field of serving people.” This definition is the most influential and dominant theoretical formulation thus far and provides research dimensions and a theoretical model for this study.

Job burnout occurs in nearly all occupations. It is very harmful to employees, their families, the service objects they serve, and even to the organizations where employees work (Simionato et al., 2019). Job burnout even has an association with an elevated risk of suicide ideation and death attempts (Freitas et al., 2023). The problem of job burnout urgently needs to be addressed, and academic circles are attempting to determine the causes of job burnout to reveal the underlying mechanism of job burnout from various perspectives. At present, there are three perspectives to explain the formation mechanism of job burnout. The view of environmental factors emphasizes that the leading cause of job burnout is environmental factors (such as job demand–resource theory (Demerouti et al., 2001)). The view of individual factors focuses on the leading causes of job burnout [such as the social competency model (Harrison, 1980), resource conservation theory (Hobfoll, 1989), and demographic characteristics (Comes and Johnson, 2017)]. The view of interaction emphasizes that individual factors and environmental factors form an interactive whole [the degree of matching between individual and work situations (Maslach et al., 2001), the perspective of the ecosystem (Gao et al., 2023), etc.]. This study agrees with the interaction view that the factors causing job burnout are complex and that it is not comprehensive to study the mechanism of job burnout from only one aspect; therefore, research should combine the two aspects of individual and environmental factors to study the mechanism of job burnout.

The theory of basic psychological needs proposes that the satisfaction of three primary psychological needs promotes the growth and perfection of an individual’s personality and cognitive structure. All individuals strive to meet these needs and tend to seek an environment for meeting these needs (Deci and Ryan, 2000; White, 1959). This theory explains the mechanism by which environmental factors affect individual behavior and mental health through the mediation of internal psychological needs (Vansteenkiste et al., 2020; Zhang et al., 2010). Some studies have shown that the theory of basic psychological needs explains individual job burnout from the perspective of interaction. The work environment affects the degree to which individuals’ basic psychological needs are satisfied; the higher the work demands are, the lower the degree to which individuals’ basic psychological needs are satisfied, and the more work resources there are, the greater the degree to which individuals’ basic psychological needs are satisfied (Van den Broeck et al., 2008). Primary psychological need frustration is highly correlated with job burnout (Ryan and Deci, 2000a,Ryan and Deci, 2000b). There is a significant negative correlation between essential psychological need satisfaction and job burnout (Liu et al., 2012); the higher the psychological need satisfaction, the lower the job burnout (Li et al., 2013), and the negative correlation between essential psychological need satisfaction and job burnout was −0.34 (Jowett et al., 2016). The frustration of basic psychological needs is positively correlated with job burnout (Bartholomew et al., 2011a,Bartholomew et al., 2011b) and has a significant positive impact on the burnout of athletes with 0.59 (Mars et al., 2017) and 0.57 (Aguirre Gurrola et al., 2016).

The satisfaction of basic psychological needs and the frustration of basic psychological needs are two independent dimensions (Deci and Ryan, 2000). The satisfaction of autonomy need refers to the experience of self-determination and full realization of self-will in carrying out activities. Autonomy need frustration leads to controlling oneself through external compulsion or self-restraint (Deci and Ryan, 1985); competence need satisfaction leads to feeling capable of achieving the desired results; and competence need frustration leads to feelings of failure and doubt about effectiveness (Chen et al., 2015). The satisfaction of relationship needs concerns intimate relationships and genuine connections with others, and the frustration of relationship needs involves the experience of exclusion and loneliness (Chen et al., 2015). The satisfaction of needs and the frustration of needs are equivalent among individuals (Chen et al., 2015), but the relationship between them is asymmetric; the satisfaction of needs can be shallow but needs are not necessarily frustrated, while need frustration means low satisfaction (Vansteenkiste and Ryan, 2013). Low satisfaction of needs is not closely related to disease but can produce long-term problems; when needs are suddenly frustrated, the worsening of discomfort is accelerated (Vansteenkiste and Ryan, 2013). Need satisfaction is more strongly associated with positive outcomes, while need frustration is more strongly related to adverse outcomes (Deci and Ryan, 2000), both of which directly affect individual motivation and functioning (Warburton et al., 2019). The satisfaction of needs supports the bright side of human functioning, while the frustration of needs drives the dark side (Rouse et al., 2019). The frustration of needs is more predictive of adverse outcomes and psychological ill health (Chen et al., 2015), which makes individuals follow external rules, and their behavior becomes uncoordinated and unmotivated. It can even lead to antisocial behavior (Deci and Ryan, 2000) and to somatization, anxiety, depression, internal contradictions, and so on (Vansteenkiste and Ryan, 2013). Olafsen et al. (2016) reported that job demand frustration was associated with higher levels of job stress, which predicted higher levels of somatic symptoms, emotional exhaustion, and absence from work due to illness. Bartholomew et al. reported that need obstruction was a better predictor of athletes’ negative behavior than need satisfaction was (Bartholomew et al., 2011a,Bartholomew et al., 2011b).

To date, a large amount of evidence has shown that primary psychological need frustration is significantly positively related to job burnout, but there is little evidence showing how need frustration affects job burnout. This study introduces the perspective of primary psychological need frustration, focusing on how it affects the occurrence and development of job burnout. It discusses the internal causal relationship between basic psychological need frustration and job burnout and explains how primary psychological need frustration affects the psychological mechanism of job burnout. The results of this study not only enrich research on the psychological causes of job burnout and the basic psychological needs of setbacks in theory but also help organizations and employees prevent and detect job burnout syndrome.

## 2 Study design

### 2.1 Research questions

This study focuses on how the frustration of basic psychological needs affects the psychological mechanism of job burnout and expounds on how individual job burnout occurs and develops through the interaction of organizational factors and individual frustration with basic psychological needs. It poses three main research questions. First, which organizational factors affect the frustration of basic psychological needs? Second, what is the result of the frustration of basic psychological needs caused by organizational factors? Third, what is the correlation between the results of primary psychological need frustration and different dimensions of job burnout?

### 2.2 Study methods

Given the purpose of the study, qualitative research methods are used to describe the participants’ emotional changes and psychological mechanisms. On the one hand, through face-to-face conversation between the researcher and the participant, the researcher develops an “explanatory understanding” of the participant’s personal experience and meaning construction, and through his own experience, the researcher explains the participant’s life story and meaning construction. On the other hand, the researcher is the research tool for studying the individual’s “life world” in a natural situation (Chen, 2000). A case study, as a qualitative research method, is used under the following three conditions: first, the main questions pertain to “how” and “why”; second, the researcher cannot control the research subject; and third, the focus of the study is the current reality (Yin, 2014). Therefore, this study uses qualitative research to explain how the frustration of basic psychological needs affects the psychological mechanism of job burnout by performing a case study of Chinese college counselors (hereinafter referred to as counselors).

### 2.3 Case selection

Counselors are the organizers, implementers, and instructors of the daily ideological and political education and management of college students, the life mentors of students’ growth and success, and intimate friends in a healthy life (PRC China, 2017). As the professional role with the most complex role structure and the heaviest responsibility in contemporary China (Dai and Xiao, 2016), the phenomenon of job burnout is widespread, and the degree of burnout is high (Guo et al., 2020; He et al., 2022; Wen et al., 2022; Zhang and Yu, 2016). Severe job burnout destroys the physical and mental health of counselors, reduces their sense of professional achievement and honor, and negatively affects the smooth development of ideological and political work in colleges and universities; at the same time, job burnout is transmitted to students through emotional rendering, which negatively impacts students’ studies, lives, and work (Huang and Wang, 2018). Therefore, the psychological mechanism of counselor job burnout is a unique and representative research topic. Zhang et al. studied 545 counselors from five colleges and universities in Heilongjiang Province, China, and found that the older counselors are, the more likely they are to experience burnout, which is consistent with findings on the job burnout of counselors who have worked for many years (Zhang et al., 2020). According to the principles of purposive sampling and convenient sampling, this study selected senior counselors with more than 13 years of service at a Chinese university as the case study. In adherence to the research ethics, the researcher informed the participants of the following study-related content in advance: research purpose, data confidentiality, voluntary participation, and voluntary withdrawal at any time without reason. They all participated voluntarily, and no one withdrew before completing the study. After the eighth interviewee was interviewed, no new content was found. Thus, the data were considered to be saturated, and data collection was considered complete. To ensure the privacy of the study case data and the confidentiality of the participants’ data, code names were assigned to the eight interviewees in place of their real names (see Table 1), including three males (represented by code M) and five females (represented by code F).

**Table 1:** Basic information on the study case.

Number	Sex	Length of service (years)	Title	Managing the number of students
M01	Male	17	Associate Professor	180
M02	Male	13	Lecturer	220
M03	Male	14	Lecturer	260
F01	Female	17	Lecturer	210
F02	Female	18	Lecturer	230
F03	Female	15	Lecturer	198
F04	Female	15	Associate Professor	209
F05	Female	16	Associate Professor	193

### 2.4 Data collection and analysis

To ensure the authenticity and validity of the first-hand data, this study conducted semi-structured interviews from May to September 2023. Each interview lasted 40–60 min, and the interview outline was prepared in advance to guide the data collection. The interview outline consisted of three open-ended questions: 1. What is the most significant difficulty or pressure you have encountered in your career? The primary sources of the respondents’ basic psychological needs and frustrations were thus obtained. 2. How do these difficulties affect you, or how do these pressures make you feel? The psychological changes in the respondents’ basic psychological needs and setbacks were thus obtained. 3. What changes have you made in your career after experiencing difficulties, or what changes have been brought to you by stress? The series of consequences caused by the frustration of the respondent’s basic psychological needs were thus identified. During the interviews, the researcher encouraged the interviewees to actively participate and raise questions, freely share their workplace experience and career experience, and reflect on and clarify the current situation of their primary psychological need frustration and setbacks at work and the resulting changes in their career psychology until both sides fully understood the emerging themes. The researcher has rich experience in qualitative interviews; uses the skills of questioning, listening, questioning, responding, interacting, observing, and recording in interviews to fully understand the true feelings of the interviewees with empathy; and is skilled at asking more in-depth questions to understand participants’ views and ideas better. Following each interview, the data were analyzed on the same day (see Table 2).

**Table 2:** Coding table of the study data.

Node	Subnode	Number of data sources	Number of reference points	Coding example
Organizational factors of basic psychological need frustration	Negative job characteristics coexist with high levels of autonomy and frustration	8	23	I really do not understand why students feel that counselors are online at work 24 h a day; that there are no time boundaries for their work; that they are required to have their cell phones on 24 h a day; and that there is no department on campus that does not call on a counselor for anything that has to do with students.
	Adverse career environments coexist with high levels of competence frustration	8	20	Identity is becoming blurred: are we lecturers, are we administrators, are we “service providers” responding to the needs of our students at all times, or are we the link between parents and the school? Why is it that we are the busiest and least understood group of people in higher education, hardly experiencing a sense of fulfillment?
	Negative behaviors of others accompany severe relationship frustration	8	16	Now the teacher–student relationship: when you need to be a teacher, [you] do not need to be a waiter, nanny or a stranger, it is difficult to remember yourself after graduation. The college leadership is angry that I do not reflect deeply, did not pay attention to this matter from an ideological point of view, so I will take the lead in the General Assembly to accept self-criticism; anything can be blamed on me. When I encountered such a leader, [I’ve been] really speechless since he took office; there has been no praise, all criticism.
The results of primary psychological need frustration	Change in motivational orientation: from uncontrolled motivation to controlled motivation	7	13	Working around appraisal indicators every day feels tiring and helpless. If you do not consider the appraisal targets in your work, not only will you be rejected by your colleagues, but your leaders will also keep an eye on you every day and always remind you to pay attention to your work duties.
	Change in work objectives: from intrinsic objectives to extrinsic objectives	6	12	Many colleagues who joined the school in the same year have long held a senior title, [while] I am still [holding an] intermediate title. I hope that my title can go up a step, with a higher salary per month, and that the low position of the school will also be raised a little.
	Behavioral pattern change: from proactive to reactive	8	17	We are also just the school to complete the work task of the tool people. Many people cannot do the main work, or in the work [there] can only be passive implementation of the instruction; there is no way to address the status quo of such a job. [One] can only try to minimize the emotional investment, isolate their inner real ideas, and maintain a certain psychological distance from the surrounding environment to reduce the internal psychological friction.
	Causally oriented change: from an autonomous style to a controlling style	6	10	Work has led me to experience a variety of things; my own mentality and character have changed a lot, and currently is in the state of no on, no go. Now work on one, according to the relevant provisions of the school and leadership requirements, according to the rules.

In this study, cluster analysis was used to identify common themes in the data, and the original data were analyzed word by word, sentence by sentence, line by line, and fragment by fragment. The text data were registered and coded with NVivo11 software. Data analysis was carried out simultaneously by three experienced researchers, and when the results of the analysis were inconsistent, they were fully discussed until a consensus was reached. Transcription data and coding at all levels were provided to the respondents, and feedback was communicated many times to ensure that the results of the study truly reflected the views of the respondents. This study used the spiral research model to classify, analyze, and organize the data systematically. According to the analytical framework of the theory of basic psychological needs, the data can be summarized into two nodes: the status of frustration of basic psychological needs and the results of frustration of basic mental needs. There are seven subnodes: the coexistence of negative job characteristics and autonomous frustration, the coexistence of a hostile professional environment and competence frustration, the coexistence of other people’s negative behavior and relationship frustration, a change in motivation orientation from non-controlled motivation to controlled motivation, a change in job goals from internal to external, a change from active to passive behavior patterns, and a change in causal orientation from an autonomous style to a controlled style.

## 3 Research results

### 3.1 Organizational factors of basic psychological need frustration

#### 3.1.1 Negative work characteristics and autonomous frustration strongly coexist

People have an innate need for a sense of autonomy, a need to feel personal causality, and a need to think that their actions are chosen by themselves, not imposed by some external source; that is, the cause of their actions exists within themselves, not in an external source. If people cannot feel autonomy, they will experience a decline in well-being, resulting in a variety of maladaptive consequences (Deci and Flaste, 2020). In this study, it is difficult for the participants to experience a sense of autonomy and meaning in their careers, which is reflected mainly in uncontrollable working hours and the authoritative obedience of bureaucratic management. First, the working hours cannot be controlled; every practitioner in the study has time anxiety, and they seldom have free time: “Work is very busy, 24 h without rest” and “heavy and trivial work, great pressure” are the typical characteristics of their work. “I do not understand why students think that counselors work 24 h online, call at night to ask about things that are not important at all, and call at noon to ask [about things]. I accept 24 h to deal with emergencies, but it does not mean that I have to do useless customer service 24 h” (M03). “I was just getting ready to go to bed at 23:00 one night when I received a call from the police saying that the boyfriend of a girl in the class had called the police because of emotional problems and that the girl was going to commit suicide. He immediately asked me to go to the scene and do ideological work. The conversation lasted until 2:30 in the morning. This sense of time’s uncontrollability and control haunts me” (F01). “There is no time boundary at work. In addition to the normal eight-hour working hours, [our] mobile phones are required to be on 24 h during non-working hours. When school or students need anything, they must be able to contact people the first time and need to be dealt with immediately, which makes it difficult for me to feel a sense of work autonomy. I feel that I am being kidnapped and controlled by work all the time” (F04). The second factor is the obedience of authority under bureaucratic management. Counselors perform more roles as managers at the bottom of the hierarchical pyramid and employ tools to achieve the goal of student management. Strict management systems, quantitative assessment indicators, and formal provisions are all based on control and coercion: “[There is] no department in the whole school that cannot order counselors, such as the Student Affairs Office, Academic Affairs Office, Security Office, School Youth League Committee, Propaganda Office, Logistics Office, etc. Even professional teachers make you go to the classroom to force students to sit in the front row to listen to lectures, the teaching staff urges you to report materials every day, the Logistics Office makes you remind students to dump garbage, and the Academic Affairs Office makes you supervise students in the classroom without food. It is all the work of counselors, and I will face all kinds of criticism if there is a slight omission so that I cannot feel the meaning of giving” (F02). “If counselors have doubts about the leadership arrangement, they will face various annual assessments every year, and the leadership will decide their future career development every minute. Students have prominent accidents at more than one o’clock in the morning at night. If they do not receive a phone call in time because they are asleep or if they do not handle the matter in time, the counselors need to be responsible for the whole incident and be investigated by the school and their families. It’s boring” (M02). “Sometimes when students have unexpected accidents, although the counselors rush to the scene the first time and try their best to deal with them, the incident is finally solved satisfactorily, but the school still has a negative evaluation of us. As long as the students have any situation, it is the counselor’s problem. This learned sense of professional helplessness and powerlessness suffocates me and makes me feel more and more that this job is meaningless” (F05) (see Table 3).

**Table 3:** High-frequency keywords of basic psychological frustration affecting job burnout.

Controlled organizational factors	Three basic psychological needs frustration	Need to frustrate results	Job burnout occurrence
Negative career characteristics	Autonomy frustration	Motivation for control: school regulations, leadership requirements	Emotional exhaustion: easy fatigue, lack of enthusiasm, distraction
Negative career environment	Competence frustration	Extrinsic objectives: title advancement, promotion in post	Depersonalization: loneliness, psychological distance, indifference to the status quo
Negative behaviors of leaders and students	Relationship frustration	Negative behaviors: poor self-control, psychological isolation Causal orientation: Controlling style	Low achievement: sense of meaninglessness, low self- evaluation and self-efficacy

#### 3.1.2 Strong symbiosis between a hostile occupational environment and competence frustration

Personality psychologist Robert White proposed that people are very eager to feel strong competence or efficiency when interacting with their environment. Competence is regarded as a basic need of human beings; that is, people may engage in various activities driven by a sense of competence to enhance their understanding of achievement (White, 1959). Everyone spontaneously wants to test themselves and explore the environment, trying to take control of the situation and affirm their abilities; when a person accepts the challenge and strives for achievement, and in their own opinion, they meet the ideal challenge, they need only to accept the meaningful personal challenge and do his best to feel competent (Deci and Flaste, 2020). In this study, the participants seldom experienced a sense of competence and achievement in their career, which is reflected mainly in the confusion regarding professional identity, the breadth of professional ability, and the dilemma of ability improvement. First, there is confusion over professional identity: “The identity of a “counselor” has gradually become blurred: is it a teacher, an administrator, a “service provider” who responds to the needs of students at any time, or a link between parents and schools? In heavy work, I will constantly have questions: the state stipulates that counselors have the dual identity of teachers and administrators, but what is the academic and professional role of counselors as teachers? As a manager, what is the management authority and scope? Why are we the busiest and least understood awkward group in colleges and universities? It’s really hard for me to experience the sense of accomplishment brought about by my career” (F01). Second, the professional ability is broad: “The scope of work of counselors is too broad, including the basic general ability (such as self-management ability, interpersonal communication ability, etc.), professional consultation ability (such as employment consultation ability, crisis management ability, etc.), and practical workability (such as ideological and political education ability, daily management ability). Counselors’ professional ability dimensions are not focused, the basic general ability is not professional, the professional consultation ability is not accurate, and the practical workability is not targeted. I do not know how to improve my professional ability to be competent for work; I feel very incompetent” (M02). Third, the dilemma of ability improvement is not only a personal embarrassment that counselors are too busy to consider but also concerns excessive external difficulties: “Work is too busy, every day is busy, time is simply not enough. There are many indicators [used] to assess counselors, including the number of dormitories, the number of lectures, conversation records, and work records. In addition, student attendance, dormitory hygiene, award evaluation rate, employment rate, rate of disciplinary violations, tuition arrears rate, failure rate, class party building, mental health, subject competitions, etc. In addition, when students fight and violate the rules, we should appear at the scene for the first time and be on duty every holiday” (M03). “There are few opportunities for counselors to go out to study and train. If the number of training places at or above the provincial level is even less, there is a drop in the bucket for each university on average. Even if we are lucky enough to have the opportunity to go out to study, the leaders of the college do not support it. They are afraid that it will be easy for students to have an accident if they are not managed. It is too difficult to rely on the support of the unit to study” (F03).

#### 3.1.3 Other people’s negative behavior and relationship frustration strongly coexist

Relationship need satisfaction refers to intimate relationships and genuine connections between individuals and others, while relationship needs frustration involves the experience of relationship exclusion and loneliness (Chen et al., 2015). Individuals’ need for relationships motivates them to engage in a variety of behaviors to increase the likelihood of being accepted and decrease the possibility of being rejected. These attempts to maximize acceptance and minimize rejection are conceptualized as efforts to maintain and enhance the value of personal relationships. Relationship value reflects the importance that others attach to building relationships with themselves. When an individual feels that their relationship value is high, they feel accepted; otherwise, they fear that he or she will be excluded and therefore tries to improve their relationship value (Burkley and Burkley, 2020). In this study, the overall feeling of the relationship value of the participants is low. More specifically, the relationship value is in an unstable state in interactions with students, while the relationship value is low in interactions with leaders and colleagues: “From the big area to the senior year, some students are absent from school, drunk, and stay out at night. They have talked all night, touched each other, and given them all hope, patience, and tolerance. “Students have said, “Teacher, you can rest assured that I will listen to you, study hard, and not make you worry about it.” I feel that my efforts will eventually be rewarded, and I experience the value and harvest of helping others at work. But in the senior year, when students go out to practice and look for jobs, if there is something to contact [the student about], some students do not reply to the message; if I urge them to complete the school task again, the student does not answer their phone, my message is not returned, or the student even directly loses contact. It is too uncomfortable” (F04). “Now [regarding] the teacher–student relationship, when you are needed, you are a teacher; when you are not needed, you are a waiter, a nanny or even an unrelated person. After graduation, it is very difficult for anyone to remember you. If you have enough disappointments and grievances, it will be very difficult for you to communicate with students frankly and unreservedly” (M01). “Last semester, I took over a class. In less than 2 months, I failed one-third of a course. The college leaders knew about it and pointed all the fingers at me for not managing the class well, and the style of study was not correct. They called me to the office and asked me how to deal with it. I said that I had a class meeting, contacted the teacher, asked the students in the class why they failed, and urged them to pay more attention to this course. College leaders said angrily that I did not reflect deeply and did not pay attention to this matter ideologically. They also asked all counselors to hold a meeting and let me take the most self-criticism at the meeting; everything can be blamed on counselors. When encountering such a leader, [I was] really speechless; since he took office, [there has been] no praise, all criticism. The employment rate is not good, the students owe fees, the examination does not pass because of the instructor’s mistake” (F05). “Between colleagues’ mutual competition, the human sentiment is light, except the essential work relationships do not include contact; they do not disturb each other mutually. They feel isolated every day” (F04).

### 3.2 Results of the frustration of basic psychological needs

Given the three essential psychological setbacks, the work motivation, work goals, behavior patterns, and causal orientation of the research subject gradually change: motivation orientation changes from non-controlled motivation to controlled motivation, work goals change from internal to external, behavior patterns change from active to passive, and the causal orientation changes from an autonomous style to a non-autonomous controlled style.

#### 3.2.1 Change in motivation orientation: from non-controlled motivation to controlled motivation

Based on differences in the degree of internalization of external rules, motivation types are divided into demotivation, external motivation, and internal motivation. There is a continuum from demotivation to intrinsic motivation in the dimension of autonomy. Demotivation is a state of unwillingness, while intrinsic motivation is a state of high autonomy and self-determination. External motivation (subdivided into external regulation, introspective regulation, identity regulation, and integration regulation) is located in the middle of the two, which is a partially autonomous control state (Ryan and Connell, 1989; Ryan and Deci, 2000a,Ryan and Deci, 2000b). Identity regulation, integration regulation, and intrinsic motivation are associated with autonomous motivation, while external regulation and introspective regulation are associated with controlled motivation (Deci and Ryan, 2008). Under autonomous motivation, behavior is accompanied by self-selection; under controlled motivation, the individual feels controlled by external forces (Deci and Ryan, 2008). In this study, the motivation orientation of the participants changed gradually from autonomous motivation to controlled motivation and even to non-motivation: “I started working with enthusiasm, and I was very happy to see the students make progress because of their hard work. After more than 10 years of work, I now meet all kinds of reasonable or unreasonable demands from students, and I will deal with them according to the requirements of the school and the leadership and the workflow to avoid being complained about by students or being held accountable by the school” (F05). “In the past, my work was very simple and happy. I felt relaxed with students, and all kinds of interpersonal relationships in my work were very simple. Now the student work competition is too fierce; the whole school student work assessment [is] to rank, the counselors’ assessment are compared. Every day, around the assessment indicators and work, [I] feel very tired and helpless. If I work according to my ideas without considering the assessment indicators, I will not only be excluded by my colleagues, but also be watched by my leaders every day, reminding me to pay attention to my duties, and will be regarded as a negative example and criticized by the General Assembly” (M01). “When I was young, I had a dream of working, and every day I thought about how to make progress in my work and improve my ability to better help students. Nowadays, the working environment of students includes rules and regulations, accountability mechanisms, and bureaucratic management. If they [counselors] are slightly negligent, they will be held accountable. Even if they work according to the requirements, they can be thrown away by schools or colleges for various reasons. Work is not interesting at all. It’s just for [a] salary to support their families” (M03).

#### 3.2.2 Change in work objectives: from internal objectives to external objectives

The work goals of individuals can be divided into two categories: external goals, including wealth, power, and status, and internal goals, including individual development, intimate relationships, and physical health (Deci and Ryan, 2000). Internal goals and external goals have different effects. The pursuit of internal goals is associated with higher well-being and good adaptability, while the pursuit of external goals is associated with lower well-being and poor adaptability; depression, anxiety, low self-esteem, and job burnout occur more often when employees value or pursue extrinsic goals more than intrinsic goals (Kasser and Ryan, 1996). In this study, the three basic psychological needs of the subjects were frustrated for a long time, the work goals changed, the weight of internal goals gradually decreased, and the weight of external goals gradually increased, developing gradually from the pursuit of internal goals to the pursuit of external goals: “When I first went to work, I had a harmonious and intimate relationship with my colleagues and students, hoping to be an excellent counselor. Now, as long as there are no omissions in the work, we should keep all kinds of traces of work and avoid being held accountable by the school. In addition, I have been working for 16 years, and many colleagues who joined the school in the same year have long been appraised with senior titles. I am still [holding] an intermediate title, and I hope that my title can be upgraded to a higher level, with a higher monthly salary and a lower level in the school” (F02). “The longer I work, the more difficult it is to work as a counselor. I had hoped that I could become an excellent expert counselor. Now, the working atmosphere of school students is too depressing, and the original intention of educating people is [being] gradually eroded by reality. Now I am thinking about how to deal with the relationship with the leaders, while leaving a good impression of excellence and competence to the leaders and hoping to have the opportunity to be promoted by the school and break away from the grassroots student work” (M01).

#### 3.2.3 Changes in behavioral patterns: from active to passive

When the three basic psychological needs are frustrated, the individual will develop a rigid and stereotyped behavior pattern to adapt to the hostile environment and protect themself from the harm caused by the blocked needs. This pattern will keep the individual from their inner experience and insist on behavior with negative results (Deci and Ryan, 2000). In this study, the behavior patterns of the participants gradually tended toward negative results: “When I first joined the work, I loved my work from the bottom of my heart and was very willing to help students. In practical work, the leaders put the task on the counselors and ordered the counselors to urge the students to complete it, the students did not respond and did not want to cooperate, and the task was not effective. The counselors were scolded by the leaders. The counselor urged the students again and again, and the students scolded the counselor. The daily work of counselors is to be scolded by leaders and students, to add endless classes, to engage in endless activities, and to promote endless work. Indeed, many things cannot be completed by students, but leadership orders must be completed. Counselors are only the tools of the school to complete a task, [yet] many things counselors cannot decide; the work can only be passively implemented. There is no way to solve such a work situation. I can only try my best to reduce my emotional investment, isolate my true thoughts, keep a certain psychological distance from the surrounding environment, and reduce internal psychological friction” (F02). “I feel that the beauty and hope of work have been gradually dispelled and [the feeling] that I love life and am full of a sense of control has disappeared, and I have become a learned helpless person. At the end of the day, my favorite thing to do is to lie on the sofa and watch videos without restraint, get a moment’s relief from self-indulgence, and then feel guilty about wasting time after watching videos” (F01).

#### 3.2.4 Causally directed change: from an autonomous to a non-autonomous controlled style

Causal orientation is a personality trait that refers to individuals’ tendency to perceive the degree of self-determination of external activities steadily. In the same environment, causal orientation can be divided into the following three types: autonomous orientation, which means that the individual is more likely to feel that the behavior is autonomous; controlled orientation, which means that the individual is more likely to think that the behavior is externally controlled; and non-individual orientation, which refers to the individual’s tendency to think that the behavior is not under their control or that they are incompetent (Zhao et al., 2016). When the individual’s basic needs are frustrated, the individual develops a non-autonomous controlled style, that is, high control motivation or no motivation (Deci and Ryan, 2000). In this study, the causal orientation of the subjects gradually became more inclined toward the non-autonomous controlled style following the long-term frustration of basic psychological needs: “After graduating from graduate school, I entered the university to do student work, every day full of blood, looking at the smiling faces of a group of young students, thinking that life is always sunny; that kind of work enthusiasm arises spontaneously from the bottom of my heart. But with time, [I] encountered a variety of things, my mentality and personality have changed a lot, the appearance of light in the eyes is gone. At present, I have been working for 17 years, belonging to the state of being unable to go up and go up. Now there is only one thing in our work, according to the relevant regulations of the school and the requirements of the leaders: we should act according to the rules, put down the complex of helping others, and reduce the internal spiritual friction” (M01). “In the past, when I encountered a student crisis, I felt very anxious and could not help breaking down. I could not control my crying when I talked about it with my family and friends. I jokingly said that I would go to the hospital to see if I was depressed. As a result, I was diagnosed with severe depression and severe anxiety after going to the hospital. For a long time, I pretended to be happy in front of people, but as soon as I mentioned the recent work content, I began to be in a bad mood and shed tears of sadness. Now I feel that my personality has become introverted, more and more concerned about the views of the people around me, less and less focused, and [that] what I am doing will be interrupted by other more urgent things at any time. Being controlled by trivial things every day consumes all [my] energy” (F03).

### 3.3 The psychological mechanism through which basic psychological need frustration affects job burnout

This study focused on the controlling environment of the participants, such as negative job characteristics, hostile occupational environment, and the negative behaviors of others. A controlled environment causes individuals to experience high frustration of basic psychological needs for a long time. The four outcomes of frustration of basic psychological needs are closely related to job burnout. The psychological mechanism by which job burnout is affected by primary psychological need frustration is manifested in four aspects: the external motivation of the research participants is highly correlated with the emotional exhaustion dimension of job burnout; the pursuit of external goals is highly correlated with emotional exhaustion and a low sense of accomplishment from job burnout; a passive behavior pattern is highly correlated with depersonalization and a low sense of accomplishment from job burnout; and the causal orientation of the participants gradually changed into a controlled style, which was the result of the long-term frustration of basic psychological needs. In turn, this change positively affected the frustration of basic psychological needs and positively predicted job burnout.

First, the motivation orientation of the research participants changed from autonomous motivation to controlled motivation or no motivation, and motivation cost increased. They experienced a lack of passion and motivation for work, the feeling of always being controlled, and emotional exhaustion. Controlled motivation is highly related to emotional exhaustion. Studies have shown that autonomous motivation significantly predicts workplace outcomes, and when people can feel the value and importance of their work, they will show enhanced work motivation (Deci et al., 2017). A survey of 806 French-Canadian teachers in public elementary and secondary schools revealed that changes in autonomous motivation negatively predicted changes in emotional exhaustion, and self-efficacy negatively predicted changes in three dimensions of job burnout (Fernet et al., 2012a; Fernet et al., 2012b). Fernet et al. (2012a; Fernet et al., 2012b) studied the autonomous motivation and control motivation of 586 school principals and found that their autonomous motivation was negatively related to work exhaustion, while control motivation was positively associated with exhaustion. Structural equation modeling of a heterogeneous sample of 370 white-collar workers in Belgium confirmed that autonomous motivation was positively correlated with overwork and that overwork was accompanied by happiness and interest or was positively correlated with vitality. Controlled motivation is positively related to compulsive work, which is accompanied by compulsion and fatigue (Van den Broeck et al., 2011). A study of 603 employees of the Portuguese Tax and Customs Authority showed that occupational stress (caused by unsupportive managers and co-workers; career and pay; and family issues) had a positive and significant effect on turnover intentions, and that this relationship was mediated by burnout. Motivation (intrinsic and determined) had a negative and significant impact on turnover intentions. Intrinsic motivation moderated the relationship between occupational stress (caused by unsupportive managers and with managers and co-workers; career and pay; and family issues) and turnover intentions (Freitas et al., 2023).

Second, the goal orientation of the research participants changed from the pursuit of internal goals to the pursuit of external goals. Although individuals will receive some benefits in the short term from the pursuit of external goals, this pursuit will lead to the destruction of one’s center self-rules and increase emotional tension (Emery et al., 2016). Although extrinsic goals are attractive, they are associated with fewer positive outcomes (such as lower job satisfaction, dedication, and energy at work) than intrinsic goal pursuit. In addition, there are more negative outcomes (i.e., greater emotional exhaustion, transient gratification following successful goal achievement, and the intention to quit) (Vansteenkiste et al., 2007).

Third, the passive behavior patterns of the research participants are evident: there is poor self-control ability at work, a low sense of self-efficacy, psychological isolation from the professional environment, indifference to the status quo that cannot be changed at work, a reduction in unnecessary emotional input, and a low sense of personal achievement. There is a sense of loneliness and a strong sense of meaninglessness because of interpersonal exclusion.

**Figure 1:**
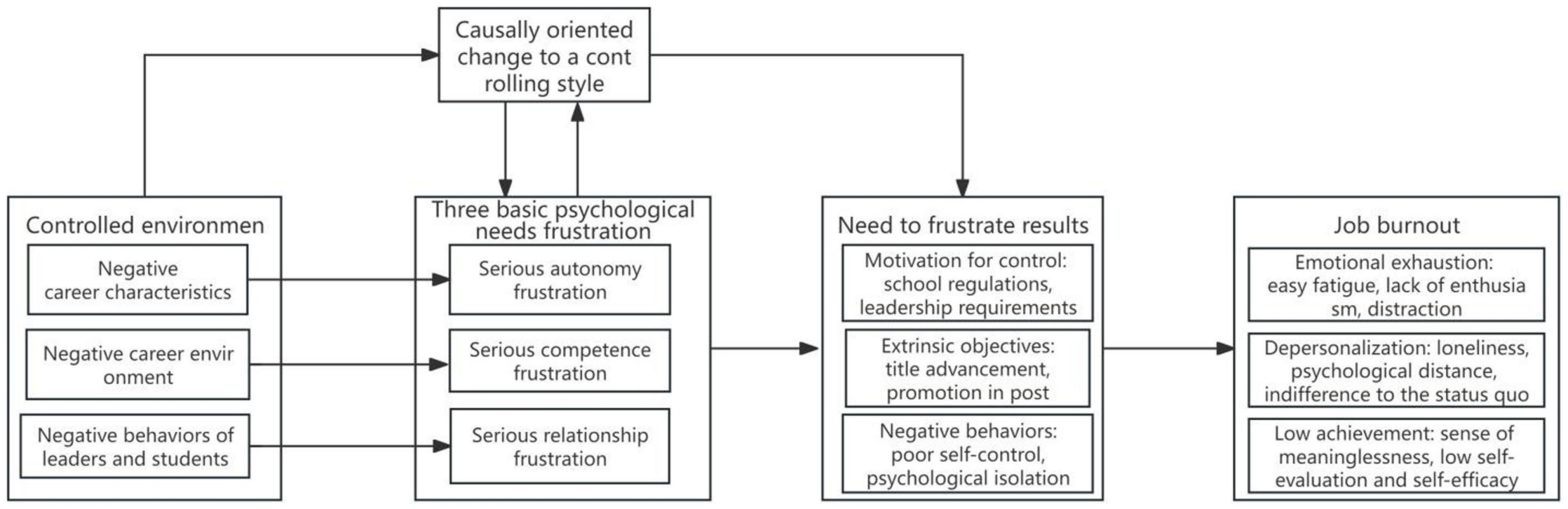
The psychological mechanism of job burnout affected by basic psychological needs and setbacks.

Fourth, as an intermediate variable, the causal orientation of the research participant gradually develops into a controlled style. The controlled style is activated in a controlled environment, which is not only the result of the long-term frustration of basic psychological needs but also positively affects the frustration of basic psychological needs and positively predicts job burnout. The results of this study on the frustration of basic psychological needs firmly explain why the participants experienced severe job burnout. The psychological mechanism through which primary psychological needs frustration affects job burnout is shown in Figure 1.

## 4 Analysis and discussion

This study focuses on how frustration with basic psychological needs affects the psychological mechanism of job burnout. The results reveal that organizational factors and individuals interact from the perspective of interaction and that high frustration with basic psychological needs affects the psychological process of the occurrence and development of job burnout. Under the background of positive psychology, this study provides strong evidence and valuable insights into the formation mechanism of job burnout.

This study revealed that the organizational factors affecting the frustration of participants’ basic psychological needs were the controlled organizational environment, such as negative job characteristics, a hostile occupational environment, and the negative behavior of others; these findings are consistent with previous studies and further confirmed the causal relationship between the controlled organizational environment and the frustration of basic psychological needs. Deci and others believe that it is essential to study how workplace factors affect the frustration of basic needs, which includes the study of various concepts of job characteristics because they represent the frustration of basic psychological needs and hinder basic psychological needs. The active obstruction of basic psychological needs in work situations is more effective in predicting adverse outcomes (such as illness) (Deci et al., 2017). The concepts of basic psychological needs for competence, autonomy, and relationships provide a framework for understanding the impact of self-determination theory on the workplace, and every policy and practice implemented within an organization may support or hinder basic psychological needs. Anyone interested in improving the organizational work environment to improve employee health can evaluate a policy or practice with the following three questions: Does it empower employees? Does the employee experience freedom of action and not feel pressured and compelled to follow instructions? Do employees have a sense of belonging to the organization? Policies or practices for which the above questions elicit positive responses are likely to promote autonomy, well-being, and quality performance, whereas those that hinder employees’ experience may promote controlled motivation or unmotivated and undesirable behavior (Deci et al., 2017).

This study revealed that the participants were in a controlled environment for a long time, and the frustration of three basic psychological needs was severe, resulting in several adverse outcomes. At present, there are three leading causes of the frustration of basic psychological needs identified in the literature: motivation costs and related direct diseases, the demand for substitutes, and the compensatory response (Vansteenkiste et al., 2020; Vansteenkiste and Ryan, 2013). The results of the essential primary psychological need frustration of the participants are consistent with the current research conclusions, which confirm the causal relationship between the controlled environment and the results of primary psychological need frustration. First, the motivation cost increases, and the motivation orientation of the research participant changes from non-controlled motivation to controlled motivation. Motivation costs associated with the frustration of basic psychological needs have been shown to include loss of motivation, disengagement, illness, and painful experiences (Vansteenkiste et al., 2020). Essential psychological need setbacks strongly predict stress (Campbell et al., 2017), anxiety (Weinstein and Ryan, 2011), and depressive symptoms (Cordeiro et al., 2016). The second is the derivation of demand substitutes, and the working objectives of the research participant change from internal objectives to external objectives. Demand substitutes are external goals that people pursue to compensate for the frustration of basic needs, such as the pursuit of prestige, status, and wealth (Kasser and Ryan, 1996; Ryan and Deci, 2000a; Ryan and Deci, 2000b). The ongoing experience of need frustration creates feelings of insecurity that lead individuals to seek external value indicators to overcome some of the insecurities and threats associated with need frustration (Ryan and Deci, 2000a; Ryan and Deci, 2000b). However, the pursuit of extrinsic goals and even the achievement of these goals are associated with reduced demand satisfaction and increased demand frustration (Leung and Law, 2019). Greater extrinsic work values than intrinsic work values have been linked to additional unpleasant outcomes, such as emotional exhaustion, higher turnover intentions, and more work–family conflict (Vansteenkiste et al., 2007). Third, compensatory behavior occurs, and the behavior pattern of the research participant changes from active to passive. Compensatory responses involve three types of behavior: release of self-control, rigid patterns of behavior, and oppositional defiant behavior (Vansteenkiste and Ryan, 2013).

This study revealed that when subjects were exposed to a controlled occupational environment for a long time, the individual’s causal quality changed, a controlled style was activated, and the causal orientation changed from autonomous to controlled. Although this finding is consistent with the current research conclusion, the literature does not find that the causal orientation change results from primary psychological need frustration. This study argues that it is reasonable to include personal causal characterization as a result of basic psychological need frustration if it develops from an autonomous style to a controlled style. When individuals are motivated by control, actions are based on the disappointment of external or internal needs, and individuals often process information in a biased, self-serving way and connect with others in a more defensive, strategic, and intolerant way. An individual’s orientation to control is a more general risk factor for defensiveness and psychopathology (Vansteenkiste and Ryan, 2013). Although autonomous and controlled styles cannot dominate individual functions, both styles can be activated by the social environment, and the direction of control is enough to trigger a defensive response, such as the use of hostile humor (Weinstein et al., 2011a; Weinstein et al., 2011b), avoidance of negative experiences (Hodgins et al., 2006), suppression of emotional distress messages (Weinstein and Hodgins, 2009), or adverse past events (Weinstein et al., 2011a; Weinstein et al., 2011b).

This study showed that primary psychological needs, frustration, and setbacks experienced by the participants led to a reduction in individual psychological energy and more severe job burnout. These outcomes manifested as emotional exhaustion (lack of passion at work, fatigue, and inattention), depersonalization (strong sense of loneliness, subconscious psychological distance), ignoring the objects of work and the status quo of work, and a low sense of achievement (strong sense of meaninglessness, low self-efficacy, low self-evaluation), which are consistent with previous research. Data from a heterogeneous sample of 3,185 Flemish employees indicated that primary psychological need frustration predicted poorer work-related well-being, which was associated with greater exhaustion (Vander Elst et al., 2012). In a heterogeneous sample of 745 workers in the Dutch-speaking area of Belgium, workers who experienced many job demands were more likely to experience primary psychological need frustration and therefore felt more exhausted (Van den Broeck et al., 2008). In a sample of 1,179 nurses in Quebec, Canada, demand-thwarting behaviors such as job bullying led to reduced job engagement and increased job burnout, which was mediated by frustration with basic psychological needs (Trépanier et al., 2013).

## 5 Conclusion

Job burnout occurs in nearly all occupations and is harmful to employees, their families, service objects they serve, and organizations. This study introduces the perspective of primary psychological needs frustration to study how basic psychological needs frustration affects the psychological mechanism of job burnout. The results showed that negative job characteristics, a hostile occupational environment, and negative behaviors of others, as controlling organizational factors, led to a higher degree of frustration in an individual’s basic psychological needs. The four types of individual primary psychological need frustration are highly correlated with different dimensions of job burnout: controlled motivation positively predicts the emotional exhaustion dimension of job burnout, the pursuit of external goals positively predicts the emotional exhaustion dimension, and the low achievement dimension, negative behavior patterns positively predict the depersonalization dimension and the low achievement dimension, and a controlling causal orientation positively predicts job burnout. From the perspective of primary psychological need frustration, this study effectively explains the psychological process of why employees experience job burnout given a controlled organizational environment and individual primary psychological need frustration. It reveals the internal causality between the frustration of basic psychological needs and job burnout, which not only enriches theoretical research on the formation mechanism of job burnout and frustration but can also help organizations and employees prevent and detect burnout syndrome in real work.

## 6 Limitations and contributions of the study

The limitations of this study are as follows. First, primary psychological frustration is influenced by personal and environmental factors. Due to the limited research ability of the researcher, this study focuses only on the controlling ecological factors of the research participants and does not explore the individual factors. Future research can enrich the understanding of the psychological mechanism through which basic psychological needs and frustration affect job burnout from the perspective of individual factors. Second, this study describes the controlled environment in detail but does not examine how to address the controlled environment, increase the internal motivation and internalized external motivation of the participants, or offer intervention methods for job burnout. Future research can explore how to provide a more positive approach to problem-solving for overcoming job burnout. Third, this study adopts a cross-sectional design. Due to the limitations of cross-sectional research methods, namely, interviews conducted at a certain time point to obtain first-hand data, there may be some degree of emotional oversight or memory selection, which may not be able to completely restore the original psychological mechanism of basic psychological frustration affecting job burnout. Future studies can avoid the limitations of cross-sectional studies by collecting more data sources and research samples at different time points.

This study makes the following three contributions. First, it explains the psychological mechanism of job burnout from the new perspective of basic psychological needs—frustration and setbacks—and supplements theoretical research on the psychological causes of job burnout. Second, most research on basic needs theory concerns the satisfaction of basic psychological needs, while less research addresses the frustration of basic psychological needs. Thus, this study supplements the existing understanding of primary psychological need—frustration—by exploring the new research field of self-determination theory. Third, the results of this study can be applied to the working environment, help individuals and organizations prevent and detect early job burnout syndrome, and enhance individuals’ occupational well-being and organizational vitality.

## Data Availability

The original contributions presented in the study are included in the article/supplementary material, further inquiries can be directed to the corresponding author.
